# Recent Advances in the Composition and Heterogeneity of the *Arabidopsis* Mitochondrial Proteome

**DOI:** 10.3389/fpls.2013.00004

**Published:** 2013-01-25

**Authors:** Chun Pong Lee, Nicolas L. Taylor, A. Harvey Millar

**Affiliations:** ^1^Department of Plant Sciences, University of OxfordOxford, UK; ^2^ARC Centre of Excellence in Plant Energy Biology, The University of Western AustraliaCrawley, WA, Australia; ^3^Centre for Comparative Analysis of Biomolecular Networks, The University of Western AustraliaCrawley, WA, Australia

**Keywords:** *Arabidopsis thaliana*, mitochondria, proteomics, heterogeneity, protein complex, post-translational modifications, functional proteomics

## Abstract

Mitochondria are important organelles for providing the ATP and carbon skeletons required to sustain cell growth. While these organelles also participate in other key metabolic functions across species, they have a specialized role in plants of optimizing photosynthesis through participating in photorespiration. It is therefore critical to map the protein composition of mitochondria in plants to gain a better understanding of their regulation and define the uniqueness of their metabolic networks. To date, <30% of the predicted number of mitochondrial proteins has been verified experimentally by proteomics and/or GFP localization studies. In this mini-review, we will provide an overview of the advances in mitochondrial proteomics in the model plant *Arabidopsis thaliana* over the past 5 years. The ultimate goal of mapping the mitochondrial proteome in *Arabidopsis* is to discover novel mitochondrial components that are critical during development in plants as well as genes involved in developmental abnormalities, such as those implicated in mitochondrial-linked cytoplasmic male sterility.

## Introduction

Mitochondria are semi-autonomous, double membrane bound organelles with unique morphologies and highly specialized functions. While these organelles are well-recognized for energy metabolism via coupling the oxidation of organic acids with oxidative phosphorylation (OXPHOS), they also have diverse functional roles such as metabolism of amino acids and biosynthesis of cofactors and vitamins. Mitochondria in plants are set apart from their mammalian counterparts by their mediation of photosynthesis through providing alternative electron sinks for photosynthetic products and their participating in photorespiration (Padmasree et al., 2002). In order to fully understand the functional roles of mitochondria in photosynthetic cells, it is essential to establish their total protein make-up (proteome) and their post-translational modifications (PTMs), as well as to generate a protein atlas that collects information about mitochondrial protein expression patterns during stress and in different cells, tissues, and organs.

*Arabidopsis thaliana* became the first model system for plants after its genome was fully sequenced and made publicly available in 2000 (The Arabidopsis Genome Initiative, 2000). In the last decade, tremendous progress has been made, by both experimental and bioinformatics approaches, to define the mitochondrial proteome in this model plant species. Like its yeast and mammalian counterparts, most of the mitochondrial proteins in *Arabidopsis* are encoded by the nuclear genome. Based on the analyses of the N-terminal targeting peptide sequences in *Arabidopsis*, there are about 2500 predicted nuclear-encoded mitochondrial proteins (representing 7–10% of all encoded proteins) with broad functional roles (Heazlewood et al., 2007; Cui et al., 2011). In comparison, the mitochondrial genome encodes for only 57 gene products (Unseld et al., 1997). The first extensive experimental studies of the mitochondrial proteome in *Arabidopsis* identified ∼100–150 proteins (Kruft et al., 2001; Millar et al., 2001; Werhahn and Braun, 2002; Millar and Heazlewood, 2003). Improvement of organelle purification procedure, availability of different protein mapping strategies, enhanced sensitivity of peptide detection by mass spectrometry (MS), and improved genomic resources and peptide identification software have driven a significant increase in the number of mitochondrial proteins identified across different model species – from 843 in *Arabidopsis* (Table S1A in Supplementary Material) and 851 in yeast (Reinders et al., 2006), to 1404 in mouse (Forner et al., 2009).

Given the number of proteins identified so far in *Arabidopsis* mitochondrion, it is clear that our understanding of its composition and functions in plants is far from complete. In this mini-review, we will provide an update on the status of *Arabidopsis* mitochondrial proteomics research based on published data in the past 5 years (2007–2012). We would refer readers to previous review articles for more comprehensive overviews on the progress of plant mitochondrial proteomics in the preceding years (Millar et al., 2005, 2011; Ito et al., 2007; Dudkina et al., 2010).

## How Far are We from Compiling the Complete Set of *Arabidopsis* Mitochondrial Proteins?

A recent in-depth analysis of the proteome in Percoll-purified mitochondria has identified a non-redundant set of 572 proteins in *Arabidopsis* cell culture (Taylor et al., 2011). With a combined proteomics, localization experiment and literature confirmation approach, a set of 38 mitochondrial proteins have been found in or associated with the mitochondrial outer membrane (Duncan et al., 2011). More recently, a total of 66 novel integral membrane proteins have been identified in mitochondria using a MS-based quantitative enrichment approach (Tan et al., 2012). A new set of components with unknown functions have also been identified in a number of recent studies, including the analysis of the mitochondrial fraction from: (i) separated protein complexes (Klodmann et al., 2010; Klodmann and Braun 2011; Klodmann et al., 2011; Schertl et al., 2012); (ii) enriched phospho-proteome (Ito et al., 2009); (iii) different tissue types (Lee et al., 2012); (iv) various time points of a diurnal cycle (Lee et al., 2010); and (v) cells subjected to biotic stress (Livaja et al., 2008).

While various large-scale proteomics studies over the last 5 years have led to the identification of a non-redundant set of 843 putative mitochondrial proteins (Table S1A in Supplementary Material), it remains difficult to discriminate true mitochondrial proteins from contaminants, particularly for low abundant proteins, in a sample. It has been estimated that about 11% of the total spot intensity on a 2-D map of mitochondria from *Arabidopsis* cell culture are proteins originated from other compartments (Taylor et al., 2011). By querying previous evidence from literature and/or consensus subcellular localization prediction score from publicly available databases [SUBA3 (Heazlewood et al., 2007); ARAMEMNON7.0 (Schwacke et al., 2003)], we define a set of 504 proteins which can be assigned to be mitochondrial-localized in *Arabidopsis* with high confidence (Table S1B in Supplementary Material). This approach is biased toward proteins that are highly abundant and does not explicitly imply that the remaining proteins are in fact contaminants from other compartments. Some of these proteins lack a predictable targeting presequence, may be dual-targeted to multiple compartments and/or are present in relatively low amount, thus their localization should be confirmed in the future through multiple independent proteomic analyses and/or by fluorescent protein localization.

According to the SUBA database, a number of GFP tagging studies have revealed the mitochondrial localization of 222 proteins that cannot be identified through proteomic approaches (Table S1C in Supplementary Material), most of which are low abundance proteins involved in the processing and maintenance of the mitochondrial genome. Together with the proteomics set, 726 proteins can be confidently assigned as mitochondrial, <30% of the presumed number of predicted proteins. To further expand the current *Arabidopsis* mitochondrial protein compendium, it is essential to overcome the challenge of identifying low abundance proteins. To achieve this a number of approaches could be employed including protein enrichment tools, such as proteominer (Fröhlich et al., 2012) or protein fractionation approaches including strong cation exchange (SCX) or off-gel electrophoresis (OGE) prior to RP-LC-MS (Chenau et al., 2008; Ito et al., 2011). Together with biological fractionation approaches such as investigation of pre-fractionated sub-mitochondrial compartments or enrichment by metal or co-factor binding approaches and advances in LC-MS techniques and equipment, it is likely that an increasing number of low abundance proteins will be revealed.

## Functions of the Mitochondrial Proteome in *Arabidopsis*

### Mitochondrial protein functions and abundance

**Figure 1:**
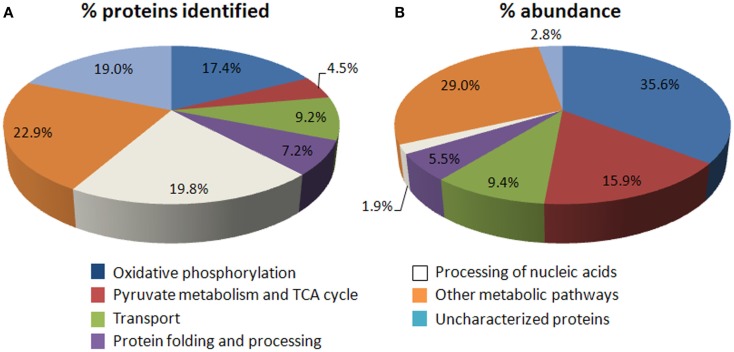
**Overview of the number and abundance of mitochondrial proteins across functional categories**. **(A)** Pie chart showing of functional categories of the confirmed set of 726 mitochondrial proteins (see Table S1B,C in Supplementary Material). A comparison with the more complete yeast mitochondrial proteome shows that similar proportion of proteins involving energy metabolism as well as proteins with unknown functions has been found (Schmidt et al., 2010). In addition, more proteins are involved in mitochondrial genome maintenance (white) in plants (∼20%) than in yeast (∼12%), due to the presence of numerous plant-specific pentatricopeptide repeat (PPR) proteins and a larger genome size. **(B)** Distribution of the abundance of proteins that can be identified by gel-free MS (Taylor et al., 2011) across seven functional categories.

Of the confirmed set of mitochondrial proteins (Table S1B in Supplementary Material), ∼22% are components of pyruvate metabolism/TCA cycle and OXPHOS, while a similar number (∼20%) are identified as subunits of machinery for mitochondrial gene expression and maintenance (Figure 1A). In the yeast mitochondrial proteome, a similar proportion (∼15%) of identified proteins are involved in energy metabolism (Schmidt et al., 2010). When comparing the abundance of proteins in these functional categories using the recently published LC-MS/MS data (Taylor et al., 2011), energy metabolism comprises over 50% of the total protein abundance in mitochondria, whereas <2% is associated with processing mitochondrial DNA/RNA (Figure 1B). The observed abundance of proteins in energy metabolism is consistent with the main role of mitochondria in the cell and bulk of the chemical reactions performed in the organelle; in contrast, the low abundance of proteins for mitochondrial DNA/RNA processing can probably be attributed to their relatively less stable nature so that they can respond rapidly to external stimuli or to changes in energy cost (Schwanhausser et al., 2011), the transient need for their functions during the life of cells and presumably the high specific activity of their functions. At the whole cellular level, components in this functional category have recently been shown to have a high turnover rate in *Arabidopsis* (Li et al., 2012). Mitochondrial proteins involving nucleic acid processing appear to perform highly specialized functions and do not seem to have overlapping specificity. Only ∼12% of the proteins in the yeast mitochondrial proteome are dedicated to genome maintenance and processing (Schmidt et al., 2010). The proportion is higher in *Arabidopsis* due to the presence of multiple plant-specific pentatricopeptide repeat (PPR) proteins and/or its larger genome size which may require more proteins to maintain and process. Each PPR protein recognizes and acts on a single site in a specific transcript sequence (Delannoy et al., 2007).

Several of the unknown proteins identified by our earlier study (Heazlewood et al., 2004) have since been re-assigned as plant-specific components of OXPHOS (Klodmann and Braun, 2011). The most nebulous subset of the known proteome is the more than 18% of the identified proteins that remain without any functional class. However, while this subset are great in number they contribute to <2% of mitochondrial protein abundance. Interestingly, these include a number of plant-specific proteins. It is therefore clear that many more studies are required to elucidate the functions of this subset of proteins which can potentially lead to the discovery of novel plant-specific mitochondrial metabolic pathways/functions.

### Protein complexes and interactome

Multiple proteins/isoforms often assembled into large complexes which serve vital metabolic and regulatory roles. While earlier reports have extensively analyzed the structure and function of individual enzyme complexes of interest, such as glycine decarboxylase complex (Douce et al., 2001), it is uncertain whether other mitochondrial proteins could also organize into macromolecular structures. Using 2-D blue-native/SDS-PAGE, Klodmann et al. (2011) found 35 different protein complexes in mitochondria from *Arabidopsis* cell culture. OXPHOS complexes are amongst the largest and the most abundant protein complexes in mitochondria. Mitochondrial complex assemblies are also dominated by components in the TCA cycle, amino acid metabolism, PPR proteins, and pre-protein import apparatus. While the preliminary compositions of these proteins complexes have been proposed based on the number of subunits identified and their migration on the first and second dimension, they must be verified through independent biochemical analysis.

A number of mitochondrial proteins of diverse function have been identified to interact with metal ions (Tan et al., 2010) and/or have binding affinity with ATP (Ito et al., 2006) in *Arabidopsis*. In contrast, studies on the more transient direct interactions (functional and physical) between multiple mitochondrial proteins in plants are lacking. Such detailed studies in the future will lead to the construction of plant mitochondrial interactome, to sit alongside side the complexome, and help to define unique metabolic regulations in plants that differentiate them from yeast and mammals.

### Post-translational modifications

The complexity of *Arabidopsis* mitochondrial proteome is further implicated by the dynamic regulation of PTMs which can control activity, stability, and structural characteristics of proteins. Proteins with PTMs often appear as multiple spots with different pI and/or molecular mass on a 2-D gel, and the region of a peptide with modified residues can be detected as an altered m/z ion species by MS. Recent large-scale proteomic studies have reported a number of PTMs in *Arabidopsis* mitochondrial proteome (Table 1), including oxidation (Tan et al., 2010; Solheim et al., 2012), phosphorylation (Ito et al., 2009; Taylor et al., 2011), S-nitrosylation (Palmieri et al., 2010), N-terminal acetylation (Huang et al., 2009), and lysine acetylation (Finkemeier et al., 2011). However, there appears to be no evidence for specific preference of PTMs to particular functional categories of identified proteins (Table 1), suggesting that PTMs have a wide variety of functional targets in the mitochondrion.

**Table 1:** **The set of mitochondrial proteins in *Arabidopsis* with known post-translational modifications**.

AGI accession	Protein ID	PTM	Reference
**OXIDATIVE PHOSPHORYLATION**			
AT1G15120	Complex III QCR6-1, Hinge protein	N-acetylation	Huang et al. (2009)
AT1G22840	Cytochrome	Lys acetylation	Finkemeier et al. (2011)
		N-acetylation	Huang et al. (2009)
AT1G47420	SDH5 succinate dehydrogenase subunit 5	Phosphorylation	Ito et al. (2009)
AT1G51980	MPP alpha-1 mitochondrial processing peptidase alpha subunit	Oxidation	Solheim et al. (2012)
AT3G12260	NADH dehydrogenase B14 subunit	N-acetylation	Huang et al. (2009)
AT3G62810	Complex 1 family protein/LVR family protein	N-acetylation	Huang et al. (2009)
AT4G21105	COX X4	Oxidation	Solheim et al. (2012)
AT5G08670	ATP synthase beta subunit	Oxidation	Solheim et al. (2012)
AT5G52840	NADH dehydrogenase B13 subunit	Phosphorylation	Taylor et al. (2011)
AT5G66760	SDH1-1 succinate dehydrogenase flavoprotein subunit	Phosphorylation	Ito et al. (2009)
ATMG01190	ATP synthase alpha-1 subunit	S-Nitrosylation	Palmieri et al. (2010)
		Oxidation	Solheim et al. (2012)
**PYRUVATE METABOLISM AND TCA CYCLE**			
AT1G24180	E1 alpha-2 (pyruvate dehydrogenase)	Phosphorylation	Ito et al. (2009)
AT1G48030	mtLPD-1 (mtlipoamide dehydrogenase-1)	S-Nitrosylation	Palmieri et al. (2010)
		Oxidation	Solheim et al. (2012)
AT1G53240	Malate dehydrogenase-1	Oxidation	Solheim et al. (2012)
At1G59900	E1 alpha-1 (pyruvate dehydrogenase)	Phosphorylation	Ito et al. (2009)
AT2G05710	Aconitate hydratase-2	Phosphorylation	Taylor et al., 2011; ref therein
		Oxidation	Tan et al. (2010)
AT2G20420	Succinyl-CoA ligase (GDP-forming) beta-chain	Oxidation	Solheim et al. (2012)
AT3G13930	E3-1 (dihydrolipoamide dehydrogenase)	Phosphorylation	Taylor et al., 2011; ref therein
AT3G15020	Malate dehydrogenase-2	Oxidation	Solheim et al. (2012)
AT3G17240	mtLPD-2 (mtlipoamide dehydrogenase-2)	S-Nitrosylation	Palmieri et al. (2010)
AT4G26970	Aconitate hydratase-1	Oxidation	Tan et al. (2010)
AT5G03290	Isocitrate dehydrogenase-5	Oxidation	Solheim et al. (2012)
**TRANSPORT**			
AT2G29530	Translocase inner membrane subunit 10, TIM10	N-acetylation	Huang et al. (2009)
AT3G08580	AAC1 (ADP/ATP carrier 1)	Oxidation	Solheim et al. (2012)
AT3G46560	Translocase inner membrane subunit 9, TIM9	N-acetylation	Huang et al. (2009)
AT5G13490	AAC2 (ADP/ATP carrier 2)	Oxidation	Solheim et al. (2012)
AT5G14040	mt phosphate transporter	Oxidation	Solheim et al. (2012)
AT5G50810	Translocase inner membrane subunit 8, TIM8	N-acetylation	Huang et al. (2009)
**NUCLEIC ACID PROCESSING AND PROTEIN FOLDING AND STABILITY**			
AT1G74230	GR-RBP5 (glycine-rich RNA-binding protein 5)	Phosphorylation	Ito et al. (2009)
AT3G13160	PPR8-2	Oxidation	Solheim et al. (2012)
AT3G23990	HSP60-3B	Oxidation	Solheim et al. (2012)
AT4G02930	Elongation factor Tu	Oxidation	Solheim et al. (2012)
AT4G26780	Co-chaperone grpE	Phosphorylation	Ito et al. (2009)
AT4G37910	Heat shock protein HSP70-1	Phosphorylation	Taylor et al., 2011; ref therein
AT5G26860	LON1 (LON protease 1)	Phosphorylation	Ito et al. (2009)
AT5G40770	Prohibitin-3	N-acetylation	Huang et al. (2009)
AT5G61030	GR-RBP3 (glycine-rich RNA-binding protein 3)	Phosphorylation	Ito et al. (2009)
**PHOTORESPIRATION**			
AT1G11860	GDT1 aminomethyltransferase	S-Nitrosylation	Palmieri et al. (2010)
		Phosphorylation	Taylor et al. (2011)
AT1G32470	GDH Glycine decarboxylase H subunit	S-Nitrosylation	Palmieri et al. (2010)
		Oxidation	Solheim et al. (2012)
AT2G35370	GDH Glycine decarboxylase H subunit	S-Nitrosylation	Palmieri et al. (2010)
		Oxidation	Solheim et al. (2012)
AT4G33010	Glycine decarboxylase P-protein 1	S-Nitrosylation	Palmieri et al. (2010)
		Oxidation	Solheim et al. (2012)
AT4G37930	SHM1 (serine transhydroxymethyltransferase 1)	S-Nitrosylation	Palmieri et al. (2010)
		Oxidation	Solheim et al. (2012)
AT5G26780	SHM2 (serine hydroxymethyltransferase 2)	Oxidation	Solheim et al. (2012)
**OTHER METABOLISM**			
AT3G61440	Cyanoalanine synthase	Phosphorylation	Taylor et al., 2011; ref therein
AT3G22200	4-Aminobutyrate aminotransferase	Oxidation	Solheim et al. (2012)
AT4G15940	Fumarylacetoacetate hydrolase family protein	N-acetylation	Huang et al. (2009)
AT5G07440	GDH2 (glutamate dehydrogenase-2)	Phosphorylation	Ito et al. (2009)
		N-acetylation	Huang et al. (2009)
		Oxidation	Solheim et al. (2012)
AT5G14780	FDH Formate dehydrogenase	Phosphorylation	Ito et al. (2009)
		Oxidation	Solheim et al. (2012)
AT5G18170	GDH1 (glutamate dehydrogenase-1)	Phosphorylation	Ito et al. (2009)
		N-acetylation	Huang et al. (2009)
AT5G50370	Adenylate kinase family	Phosphorylation	Taylor et al. (2011)
		N-acetylation	Huang et al. (2009)
AT5G63400	ADK1 Adenylate kinase 1	N-acetylation	Huang et al. (2009)
**PROTEINS WITH UNKNOWN FUNCTIONS**			
AT2G39795	mt glycoprotein family protein	Phosphorylation	Ito et al. (2009)
AT3G18240	Unknown protein	Phosphorylation	Ito et al. (2009)
AT3G55605	Mitochondrial glycoprotein family protein	Phosphorylation	Ito et al. (2009)
AT4G21460	Unknown protein	Phosphorylation	Ito et al. (2009)
AT4G27585	Stomatin-like protein	Phosphorylation	Ito et al. (2009)
AT4G23885	Unknown protein	N-acetylation	Huang et al. (2009)

*PTM, post-translational modification(s)*.

The total number of identified proteins with PTMs is very likely a gross underestimation due to a number of technical challenges, such as the loss of PTMs during mitochondrial purification procedures and the relatively low abundance of the modified peptides compared to their unmodified counterparts. Also, it is not clear how many proteins, including those listed in Table 1, are functionally modified through enzyme-catalyzed mitochondrial processes *in vivo*. For example, degradation products observed on a 2-D gel often perceive as artificial post-purification events. These concerns can be at least partially overcome by enrichment of modified peptides/proteins and/or repeat analysis of multiple replicates to ensure that similar changes can be observed in all samples. Alternatively, the incorporation of radioactive tracers into proteins *in vivo* (cells) or *in vitro* (isolated mitochondria) can be used to identify proteins with reversible PTMs. For instance, 18 phosphoproteins have recently been identified by [γ^32^P]-ATP labeling and affinity enrichment of isolated mitochondria (Ito et al., 2009).

## Changes in the Mitochondrial Proteome in Different Tissues and in Response to Oxidative Stress

The mitochondrial proteome is not static, but has many components that are dynamically regulated in order to meet energy and metabolic needs required by the cell in response to developmental and/or environmental changes. There are many different cell/tissue/organ types which have functions that are unique to plants. Thus, mitochondrial composition, metabolism, and stress response in these cells/tissues/organs from *Arabidopsis* will be different from what has been observed in yeast and animals.

Analysis of the mitochondria proteome from photosynthetic shoots, non-photosynthetic cell culture, and roots identified major differences in the abundance of enzymes of the TCA cycle and photorespiration (Lee et al., 2008, 2011). Quantitative comparison of the mitochondrial proteome across 10 different time points covering 24-h of the life of *Arabidopsis* shoots also uncovers day (photosynthetic)- and night (non-photosynthetic)-enhanced proteins in central carbon metabolism (Lee et al., 2010). In these studies, the abundances of OXPHOS complexes in purified mitochondria generally remain unaltered but their respiratory capacity differs depending on the choice and/or availability of substrates (Lee et al., 2008, 2011). However, on a whole tissue basis differences in mitochondrial electron transport chain complex ratios between tissues has been reported (Peters et al., 2012). Lee et al. (2012) have reported changes in the isolated *Arabidopsis* mitochondrial proteome beyond differences in the cellular photosynthetic capacity. Changes in the abundance of a wide variety of mitochondrial proteins can be observed from cells/tissues from various vegetative and reproductive phases of development. Differences in protein accumulation and metabolic specializations of these mitochondria generally coincide with the main physiological role of each corresponding tissue type, such as glycine cleavage via photorespiration in shoot and maintenance of mitochondrial redox environment in flowers. In mouse, it has been reported that just over half of all proteins identified by gel-free MS approach can be found in all the investigated organs (Pagliarini et al., 2008). However, the number of mitochondrial proteins that are highly tissue-specific (i.e., totally absent in at least one tissue) in *Arabidopsis* remains to be defined. Such analysis will assist in identifying mitochondrial components that causes plant-specific developmental phenotypes, e.g., cytoplasm male sterility.

Using a gel-free quantitative MS approach, Tan et al. (2012) recently identify a number of integral membrane proteins in mitochondria that are altered in abundance in response to cold and/or various chemical stresses. These proteins include the components of the alternative NADH dehydrogenases, alternative oxidase, and uncoupling proteins, but also several stress-sensitive subunits within the OXPHOS complexes. Together with a similar study by Sweetlove et al. (2002), it is concluded that the reduction in respiration in response to chemical-induced oxidative stress is a consequence of coordinated changes in the mitochondrial proteome, particularly OXPHOS complex subunits and stress-related components.

## Application of Proteomics to Analyze Mitochondrial Protein Functions

Over the last decade, advances in the understanding of mitochondrial composition and protein complex assembly have led to the identification of many genes associated with genetic diseases in humans (Calvo and Mootha, 2010). In contrast, plant proteomics still needs to discover novel mitochondrial components that are associated with known developmental defects in plants. Nevertheless, by combining proteomics and reverse-genetics strategies, a number of recent studies have highlighted the unique role of a mitochondrial component of interest in *Arabidopsis* that had not been unraveled by other biochemical and molecular techniques. Metabolite analyses of malate dehydrogenase (MDH) antisense and knockout lines in tomato and *Arabidopsis* respectively show an elevated foliar ascorbate level (Nunes-Nesi et al., 2005; Tomaz et al., 2010). Such accumulation coincides with the reduction of Complex I-associated galacton-1,4-lactone dehydrogenase (GLDH) abundance in the mitochondrial proteome of a MDH double mutant (*mmdh1mmdh2*; Tomaz et al., 2010), indicating that there might be a complex metabolic regulation/interaction between OXPHOS, TCA cycle, and cellular ascorbate biosynthesis. A mutation in mitochondrial Lon protease leads to a retarded growth phenotype (Rigas et al., 2009), which can be explained by an altered abundance of enzymes in the TCA cycle and OXPHOS, a decrease in the abundance of breakdown products and a small increase in the number of proteins with oxidized peptides, but not by heightened oxidative stress (Solheim et al., 2012). In contrast, knockout of the protease AtFtsH4 does not significantly affect *Arabidopsis* growth under long day conditions, but changes rosette development under short-day conditions (Gibala et al., 2009). The phenotypes correlate with elevated levels of oxidative stress, increased abundance of Hsp70 and prohibitins, and decreased abundance of ATP synthase subunits.

## Conclusion and Perspectives

The availability of the full genome sequence of *Arabidopsis* for more than a decade, advances in various proteomic technologies, as well as their wider adoption, have provided an opportunity to understand the protein make-up of mitochondria and their underlying metabolism in this plant more than in any other. Significant progress in extracting information on PTMs and protein abundances has also improved our insight into the dynamic regulation of the mitochondrial proteome in a cellular/organismal context. However, further work is needed to characterize mitochondrial proteins according to their sub-organellar localization. In-depth identification of components in the intermembrane space has not been reported since the improvements in MS analysis in recent years. Recent discoveries of a pyruvate transporter (Herzig et al., 2012) and a calcium uniporter (Baughman et al., 2011) in mouse mitochondria have been conducted through an integrated proteomics, bioinformatics, and genetics strategy. Thus, identification of low abundance proteins should allow us to complete the catalog of mitochondrial proteins in *Arabidopsis*, which will provide us several candidates for identifying plant-specific transporters or metabolic pathways by a similar approach.

## Conflict of Interest Statement

The authors declare that the research was conducted in the absence of any commercial or financial relationships that could be construed as a potential conflict of interest.

## Supplementary Material

The Supplementary Material for this article can be found online at http://www.frontiersin.org/Plant_Proteomics/10.3389/fpls.2013.00004/abstract

Supplementary Table S1 **Mitochondrial proteins identified by mass spectrometry or fluorescent protein localization studies**. **(A)** All proteins identified from isolated mitochondria from *Arabidopsis* using proteomics in the last 5 years. **(B)** A set of proteins which has a high probably being located in the mitochondrion. For the inclusion of a protein in the list, it should meet the following criteria: (i) A protein is automatically considered mitochondrial if at least two studies have identified it in isolated mitochondrial fraction. However, a protein is considered to be non-mitochondrial if it is identified in equal number of or more non-mitochondrial proteomics studies than the mitochondrial ones. (ii) If the location of a protein is verified independently by fluorescent protein localization analysis, then (ii) is ignored and it is included in the list. (iii) If a protein is identified by one study, the localization based on SUBAcon score and/or ARAMEMNON localization consensus score is also considered. **(C)** Mitochondrial protein confirmed through fluorescent protein localization studies (according to SUBA) only and not by proteomics.
